# Thermal Load and Heat Transfer in Dental Titanium Implants: An Ex Vivo-Based Exact Analytical/Numerical Solution to the ‘Heat Equation’

**DOI:** 10.3390/dj10030043

**Published:** 2022-03-10

**Authors:** Grigorios P. Panotopoulos, Ziyad S. Haidar

**Affiliations:** 1 Departamento de Ciencias Físicas, Universidad de la Frontera, Temuco 4811186, Chile; grigorios.panotopoulos@ufrontera.cl; 2 BioMAT’X I+D+i (Haidar Lab), Universidad de los Andes, Santiago 7550000, Chile; 3 Centro de Investigación e Innovación Biomédica (CiiB), Universidad de los Andes, Santiago 7550000, Chile; 4 Programa de Doctorado en BioMedicina, Facultad de Medicina, Universidad de los Andes, Santiago 7550000, Chile; 5 Facultad de Odontología, Universidad de los Andes, Santiago 7550000, Chile

**Keywords:** dental implants, thermal stress, modeling of heat transfer, temperature changes, heat equation, analytical solution

## Abstract

Introduction: Heat is a kinetic process whereby energy flows from between two systems, hot-to-cold objects. In oro-dental implantology, conductive heat transfer/(or thermal stress) is a complex physical phenomenon to analyze and consider in treatment planning. Hence, ample research has attempted to measure heat-production to avoid over-heating during bone-cutting and drilling for titanium (Ti) implant-site preparation and insertion, thereby preventing/minimizing early (as well as delayed) implant-related complications and failure. Objective: Given the low bone–thermal conductivity whereby heat generated by osteotomies is not effectively dissipated and tends to remain within the surrounding tissue (peri-implant), increasing the possibility of thermal-injury, this work attempts to obtain an exact analytical solution of the heat equation under exponential thermal-stress, modeling transient heat transfer and temperature changes in Ti implants (fixtures) upon hot-liquid *oral* intake. Materials and Methods: We, via an ex vivo-based model, investigated the impact of the (a) material, (b) location point along implant length, and (c) exposure time of the thermal load on localized temperature changes. Results: Despite its simplicity, the presented solution contains all the physics and reproduces the key features obtained in previous numerical analyses studies. To the best of our knowledge, this is the first introduction of the intrinsic time, a “proper” time that characterizes the geometry of the dental implant fixture, where we show, mathematically and graphically, how the interplay between “proper” time and exposure time influences temperature changes in Ti implants, under the suitable initial and boundary conditions. This fills the current gap in the literature by obtaining a simplified yet exact analytical solution, assuming an exponential thermal load model relevant to cold/hot beverage or food intake. Conclusions: This work aspires to accurately complement the overall clinical diagnostic and treatment plan for enhanced bone–implant interface, implant stability, and success rates, whether for immediate or delayed loading strategies.

## 1. Introduction

Despite significant progress in the diagnosis, prevention, management, and treatment of oro-dental diseases, teeth and supporting tissues, either damaged or lost due to disease or trauma, continue to embody a burden. The quality of life of men and women of all ages worldwide is affected by a missing tooth in several ways. Speaking difficulties, pain, loss of confidence, and poor eating (chewing/mastication/deglutition) capacity, are only a few aspects of this. Thus, a reduction in number of teeth may deteriorate quality of life (QoL) [1]. Moreover, several previously-reported articles stated that missing teeth are closely related to death [2,3,4]. Nowadays, a number of options exist for the replacement of missing teeth. Dental implants are used to replace missing, lost, or extracted teeth [5], a great option for patients missing (partially or fully) natural teeth/dentition, because they act as a secure anchor for artificial replacement teeth and/or prosthetics and eliminate the instability associated with surface adhesives and removable bridges [6]. Hence, dental implants, Titanium-based mainly, are a predictable treatment option/modality for the rehabilitation of partially- and completely-edentulous patients. Further, when compared to traditional bridges and dentures (removable solutions), implant-retained prostheses (fixed solutions) might tend to be easier to maintain and could require fewer re-visits to the dental clinic. Indeed, dental implant use has nearly tripled since its introduction in 1986 [7], and it is expected to continue to rise or grow, rapidly. People of all ages are turning to dental implants to replace a single tooth, several teeth, or a full set of dentures. The leading reasons for choosing or preferring dental implants are: to restore normal mastication/eating (and choice of foods), speaking and laughing, to enhance facial appearance, smile and confidence (increase self-esteem and reduce self-consciousness), and to increase denture retention (via improving the support to facial muscles). Dental implants changed (and continue to change) the way people live; they are re-discovering the comfort and confidence to chew, eat, speak, smile, laugh, socialize, and enjoy life overall; indeed, this has a positive impact on QoL [6].

Due to consumption of hot foods and liquids, the human tooth is daily subjected to thermal loading. Heat generated on the tooth surface from intra-oral temperature changes is transferred via conduction through the enamel, dentin, and pulp. Since enamel and dentin have lower values of thermal conductivity, the pulp is protected against rapid thermal fluctuations [8]. The thermal behavior, however, of restored teeth is significantly different in comparison to intact teeth, as the metals used in clinical restorative applications, such as titanium or titanium alloy, are excellent thermal conductors [9,10,11,12,13,14]. High temperatures may cause irreversible damage to tissues and organs [15], while the habitual consumption of extremely hot foods and beverages may affect implant treatment modality. Mechanical stability of dental implants is a prerequisite for successful rehabilitative and restorative therapy, and furthermore, it can be stated that the cornerstone of successful dental implant therapy is an intact biological osseointegration around the implant (fixture), thereby playing an important role in provision of the pursued stability. Osteoblast cells require in situ activation to increase bone density and establish high anchorage and subsequent high stability, survival, and success of the implant [16]. Thermal injury to the implant–bone interface may lead to bone necrosis and loss of osseointegration. Previous studies have shown that osteoblasts may be severely damaged by a thermal impulse of 42 degrees (10-minute heat shock) [17], and that some bone proteins are lost [18]. Furthermore, it was stated that the temperature threshold for necrosis of the bone (cortical) is 47 degrees (for 1 min) [19,20,21]. Yet, the literature reports intra-oral temperatures reaching 67–77 degrees during function and during the consumption of hot water/liquids [22,23].

Intra-bony heat generation, during surgical implant insertion, is another story (alarming), with few serious reports on temperatures at the implant–bone interface, whether during and post-surgical preparation, and/or during and post-hot substance consumption. Questions pertinent to threshold level(s) and probable transient changes in osteoblasts are raised. Thus, the transient heat transfer under thermal load is of *vital* significance in dentistry, in general, and in practical oro-dental implantology, in particular. In the literature, there already exist some previous works on the subject, where the authors attempted to model and investigate the effects of “thermal load(s)” on the bone–implant interface system [24,25,26,27,28] (see, however, e.g., [29,30] for heat transfer from warm water to foot in a footbath). In this work, our approach is distinct in two main respects: (**1**) firstly, in the other studies, authors tend to solve the heat equation with a source term that accounts for the temperature excess T-Ts, with (T) being the temperature at a given point and (Ts) being the temperature from the surroundings. Herein, we find it more natural to integrate the heat equation without the additional source term and opted to take into account the thermal load into the imposed boundary conditions; and (2) secondly, in other works, the investigation is often based on a typical or traditional numerical analysis (approach), and, to the best of our knowledge, an exact analytical solution is still missing. Obtaining an exact analytic expression for the solution is always challenging and desirable, since the physics are more transparent, while at the same time, being accessible to everyone. In particular, the interested reader may use the analytic expression either to check/reproduce the results shown in articles or to study other aspects of the solution not considered in publications.

Therefore, in the present work, our goal is two-fold: (**A**) We propose to solve the standard heat equation, modifying the imposed boundary conditions without any additional source term, and on the other hand, (**B**) we fill a gap in the literature via obtaining an exact analytical solution of a somewhat simplified problem, which nevertheless, encapsulates the physics and reproduces the results already found in previous works via numerical analyses. In addition, for the first time, we introduce and involve herein, the intrinsic time, a “proper” time that characterizes the geometry of the dental implant fixture and overall system, and we show how the interplay between that time and the exposure time influences temperature changes, and subsequent implant survival. 

Thus, this work aims to complement the overall clinical diagnostic and treatment plan for enhanced biological bone–implant interface and mechanical implant stability and success rates, whether for immediate or delayed implant loading strategies.

To simplify the flow, the plan of our work is narratively, as follows: Following the problem formulation, we obtain an analytical solution, followed by the appropriate figures (numerical results) demonstrating its main features. Two appendices are included for self-completeness and to avoid interrupting the flow of the discussion.

## 2. Thermal Load and Heat Transfer

### 2.1. Formulation of the Physical Problem

**Figure 1:**
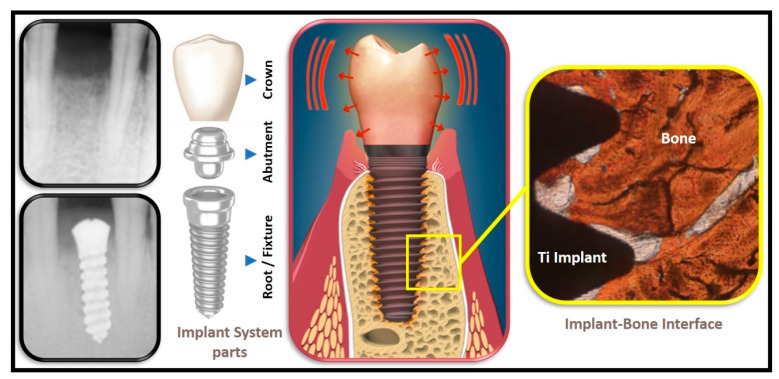
Oro-Dental Implantology, illustrating the main parts of a dental implant system and the implant–bone interface.

The dental implant system, typically, consists of three main parts, namely the root or fixture (good conductor), the abutment (good conductor) as well as the crown (moderate to poor conductor) (see Figure 1). In principle, one must solve the full three-dimensional/3-D heat equation for the temperature *T*(*t*,*x*,*y*,*z*):

(1) $$\frac{\partial T}{\partial t}=\alpha \left(\frac{\partial^{2}T}{\partial x^{2}}+\frac{\partial^{2}T}{\partial y^{2}}+\frac{\partial^{2}T}{\partial z^{2}}\right)$$

where *α* is the thermal diffusivity, which is assumed to be a constant, space, and temperature independent. For a derivation of the heat equation, the interested reader may consult Appendix A. An additional source term q(t) = m (T-Ts), where m is a constant depending on the type and the geometry of the implant, may be added to take into account the temperature excess due to the thermal loading [25,26]. 

Here, however, as already mentioned in the introduction, we propose to solve the standard heat Equation (1) and consider thermal stress via imposing the appropriate boundary condition (see the discussion below).

Given the geometry of the implant, it may be modeled as a cylinder with length L and radius R, and so one expects to obtain an axisymmetric solution where the temperature will not depend on the rotation angle, and therefore, the problem is essentially two-dimensional. During heat transfer along the axis of the implant, heat loss occurs in the radial direction as well. In the present work, however, since we are mostly interested in temperature changes along the axis of the implant, we shall ignore the radial dependence, and thus we shall solve the one-dimensional heat equation (an approximation also justified by the fact that typically the length is considerably larger than the radius of the implant) for the temperature *T*(*t*, *y*):

(2) $$\frac{\partial T}{\partial t}=\alpha \frac{\partial^{2}T}{\partial y^{2}}$$

in the domain *t* > 0, and 0 < *y* < *L*, assuming for simplicity a single value of the thermal diffusivity throughout the length of the implant, corresponding to that of the abutment. It is perhaps note-worthy herein that since the implant consists of three (main) parts, each with its own thermal diffusivity, in a more realistic analysis, one may consider a piece-wise function (i.e., a constant value in each individual part, which is different than the other two); yet, it would not be possible to then write down a tractable solution.

In this work, we shall consider two numerical values between the diffusivity of titanium and that of ceramic. The endpoint *y* = 0 corresponds to the bone, while the other endpoint *y* = *L* corresponds to the oral cavity.

This partial differential equation must be supplemented with the initial condition *T*(*t* = 0, *y*) = f(*y*) as well as with the two boundary conditions at the surface (oral cavity) and at the bone, namely *T*(*t*, *y* = *L*) = *T_surface_*(*t*) and *T*(*t*, *y* = 0) = *T_bone_*(*t*), where the functions *T_bone_*(*t*), *T_surface_*(*t*), and f(*y*) are given functions depending on the physics of the problem at hand. This problem is well posed, and it has a unique solution [31]. For example, in the simplest case, in which

(3) $$T\left(t=0,y\right)=T_{0}$$

(4) $$T\left(t,y=L\right)=T_{0}$$

(5) $$T\left(t,y=0\right)=T_{0}$$

where *T*_0_ is a constant temperature (e.g., room temperature or body’s natural temperature) the unique solution must be *T*(*t*, *y*) = *T*_0_, as it clearly satisfies the heat equation and all conditions.

In the present work, we are interested in studying the effect of thermal stress, where it is assumed that the temperature of the oral cavity starts from, a high temperature *T*_1_ = 60 degrees, and then it decreases monotonically until it eventually reaches the body temperature *T*_2_ = 37 degrees. 

**Figure 2:**
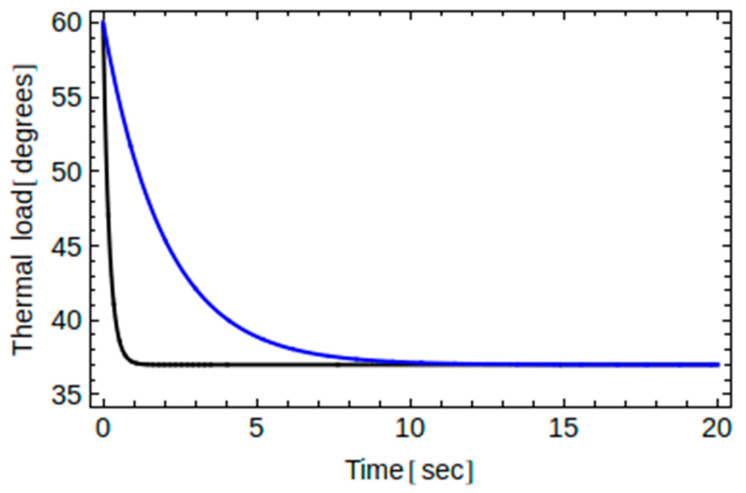
Thermal load for two different exposure times, *t*_0_ = 0.2 s (black color) and *t*_0_ = 2 s (blue color). The first one drops faster.

We can model this behavior in a simple and at the same time realistic way, introducing an elementary function exhibiting a smooth and continuous transition from *T*_1_ down to *T*_2_. In particular, in the discussion to follow, we shall consider an exponential function as follows:

(6) $$T_{oralcavity}\left(t\right)=\left(T_{1}-T_{2}\right)exp\left(-t/t_{0}\right)+T_{2}$$

where *t*_0_ is the thermal stress exposure time that shows how fast the temperature of the oral cavity drops to the body’s natural temperature (Figure 2). In fact, if we take *t*_0_ = 0.2 s, which corresponds to a duration of approximately 1 s, or *t*_0_ = 2 s, which corresponds to a duration of 10 s, our thermal loading resembles the ones commonly considered in previous works [21,24,26,27,28,29,30]. Therefore, in what follows, we assume an initial condition *T*(*t* = 0, *y*) = *T*_2_, a boundary condition at the bone *T*(*t*, *y* = 0) = *T*_2_, and a boundary condition at the surface *T*(*t*, *y* = *L*) = *T_oralcavity_*(*t*) given in (8), and we define Δ*T* = *T*_1_ − *T*_2_ = 23 degrees.

### 2.2. Exact Analytical Solution of the Initial/Boundary Value Problem

We now proceed to find the solution of the one-dimensional heat equation with the above initial and boundary conditions. Since the boundary conditions are non-homogeneous, we employ the standard trick by writing *T*(*t*, *y*) as a sum of two functions

(7) T(t,y)=u(t,y)+w(t,y)

where *w*(*t*, *y*) is a particular function that absorbs the non-homogeneous boundary conditions, so that the second function *u*(*t*, *y*) satisfies homogeneous boundary conditions. It is easy to verify that the function *w*(*t*, *y*) is given by:

(8) $$w\left(t,y\right)=T_{2}+\frac{\Delta T}{L}yexp\left(-t/t_{0}\right)$$

while *u*(*t*, *y*) satisfies the following partial differential equation:

(9) $$\frac{\partial u}{\partial t}-\alpha \frac{\partial^{2}u}{\partial y^{2}}=\frac{\Delta T}{Lt_{0}}yexp\left(-t/t_{0}\right)$$

and the conditions

(10) $$u\left(t=0,y\right)=-y\frac{\Delta T}{L}$$

(11) u(t,y=L)=0

(12) u(t,y=0)=0

We see that the price to pay is that *u*(*t*, *y*) satisfies a non-homogeneous differential equation. However, this does not pose a problem since we can make the following ansatz by expanding on a complete basis of functions:

(13) $$u\left(t,y\right)=\overset{\infty }{\underset{n=1}{\sum }}C_{n}\left(t\right)sin\left(\frac{ny}{L}\right)$$

where *n* = 1, 2, … is an integer, and *Cn*(*t*) are unknown functions of time depending on *n*. The homogeneous boundary conditions at *y* = 0, *L* are automatically satisfied, while the coefficients *Cn*(*t*) can now be determined by using the differential equation and the initial condition. First of all, we use the fact that any function *g*(*y*) can be expanded on the basis sin(*n* π *y*/*L*) in the following form:

(14) $$g\left(y\right)=\overset{\infty }{\underset{n=1}{\sum }}A_{n}sin\left(\frac{n\pi y}{L}\right)$$

with coefficients *A_n_* that are given by:

(15) $$A_{n}=\frac{2}{L}\overset{L}{\underset{0}{\int }}dyg\left(y\right)sin\left(\frac{n\pi y}{L}\right)$$

using the orthogonality of the basis:

(16) $$\overset{L}{\underset{0}{\int }}dysin\left(\frac{n\pi y}{L}\right)sin\left(\frac{m\pi y}{L}\right)=\frac{L}{2}\delta_{n,m}$$

where *δn*,*m* is the Kronecker symbol taking the value 1 when *n* = *m* and 0 otherwise. For the function *g*(*y*) = *y*, the coefficients are given by:

(17) $$A_{n}=\frac{{\left(-1\right)}^{n+1}2L}{\pi n}$$

The initial condition implies *Cn*(0) = −Δ*T A_n_*/*L*, while if we plug the ansatz into the partial differential equation, we obtain an ordinary differential equation for *Cn*(*t*) as follows:

(18) $$\frac{dC_{n}\left(t\right)}{dt}+\alpha {\left(\frac{n\pi }{L}\right)}^{2}C_{n}\left(t\right)=\frac{\Delta TA_{n}}{Lt_{0}}exp\left(-t/t_{0}\right)$$

In Appendix B, we describe how to obtain the solution to the above differential equation. Putting everything together, and defining *τ*= $L^{2}$/(*α* $\pi^{2}$) to be the intrinsic time of the dental implant, the exact analytical solution to the initial/boundary value problem is given by:

(19) $$T\left(t,y\right)=T_{2}+\frac{\Delta T}{L}yexp\left(-t/t_{0}\right)+\frac{2\Delta T}{\pi }\overset{\infty }{\underset{n=1}{\sum }}D_{n}\left(t\right)sin\left(\frac{n\pi y}{L}\right)$$

(20) $$D_{n}\left(t\right)=\frac{{\left(-1\right)}^{n+1}}{n}\left[\frac{exp\left(-t/t_{0}\right)}{-1+n^{2}\left(t_{0}/\tau \right)}-\left(1+\frac{1}{-1+n^{2}\left(t_{0}/\tau \right)}\right)exp\left(-n^{2}t/\tau \right)\right]$$

We notice that the solution does not depend on *L* and *α* separately, but only through the intrinsic time *τ*. It is easy to check that the initial and boundary conditions are satisfied. Additionally, we see that the above solution approaches the natural temperature of the body after a sufficiently long time, *T*(*t*, *y*) → *T*_2_ as *t* →∞.

This is to be expected, since the temperature of the oral cavity eventually drops to the body’s natural temperature, and from that moment on, the unique solution of the heat equation with constant initial/boundary conditions corresponds to the constant temperature *T*(*t*, *y*) = *T*_2_. As we will see shortly, the temperature changes versus time at a certain location, e.g., close to the surface or close to the bone, depend on the interplay between the exposure time *t*_0_ and the intrinsic time of the implant *τ* = $L^{2}$/(*α* $\pi^{2}$), which, for a given length *L*, is low for good thermal conductors and high for poor conductors.

We remark that in works studying thermal therapies, a very important concept is that of thermal dose. First described by Sapareto and Dewey [32], it is computed cumulatively using an empirical formula, and in practice, it works as follows: for every degree above 43 degrees, the required time to coagulate the tissue halves, i.e., 120 min at 44, 60 min at 45, etc. For more details, please also see [33].

For the length considered here (*L* = 1.3 cm, see next section), for Titanium, the intrinsic time is found to be *τ* = 1.9 s, while for ceramic, it is computed to be *τ* = 27.4 s (Appendix A).

## 3. Main Features of the Solution

Herein, we attempt to demonstrate, in a pictorial way, the behavior and main features of the exact analytical solution obtained in the previous section. This is accomplished through a series of figures, in which we demonstrate/show temperature changes versus time for (i) two types of implants A and B; (ii) several different numerical values of the exposure time *t*_0_; and (iii) location point along the axis of the implant. Our approach is theoretical, with no experimental validation feasible or at disposal. Nonetheless, the figures below clearly show that all key features observed in the earlier related studies are reproduced.

The system, in principle, is characterized by three free parameters, namely the length of the implant *L*, the thermal diffusivity of the material *α*, and the exposure time *t*_0_ of the thermal load, and not on characteristics such as age, sex, etc., of the patients. In practice, however, the behavior of the solution depends on the interplay between *t*_0_ and the intrinsic time of the implant *T* (please see discussion below).

$$\tau =L^{2}/\left(\alpha \pi^{2}\right)$$

In the following, we find it natural to split the free parameters in two ways, as follows: (*i*) on one hand, the duration of the load, roughly 5 *t*_0_; and (*ii*) on the other hand, the intrinsic time *τ* of the geometry of the dental implant. The length of the dental implant varies from 7 mm to 20 mm [24]. Here, we fix the length at *L* = 1.3 cm as in [25]. Moreover, for a given geometry and a certain thermal load, temperature changes depend on the location along the implant. Following the standard notation, we introduce the location points B1 at *y* = 3 *L*/4 (superficial), B2 at *y* = *L*/2 (middle), and B3 at *y* = *L*/4 (deep) [26]. Finally, we consider two different types of implants, type A with *α* = 2 × $10^{-6}$($\begin{array}{c} m^{2} \end{array}$/s) (moderate conductor), and type B with *α* = 5 × $10^{-6}$($\begin{array}{c} m^{2} \end{array}$/s) (good conductor), with values comparable to those employed in [25,26]; consequently, our approach and findings may be directly compared to the results obtained therein.

**Figure 3:**
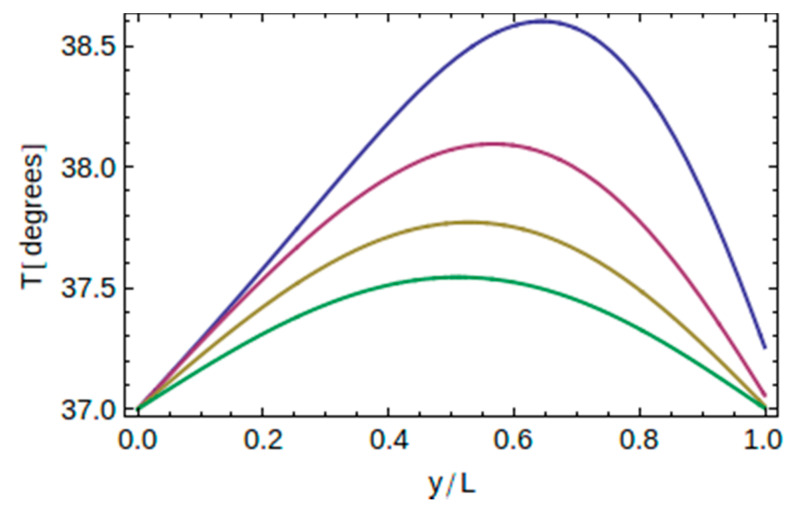
Temperature distribution (in degrees °C) versus location at four different instants of time (from top to bottom 9 s, 12 s, 15 s, and 18 s) for implant A and for *t*_0_ = 2 s.

**Figure 4:**
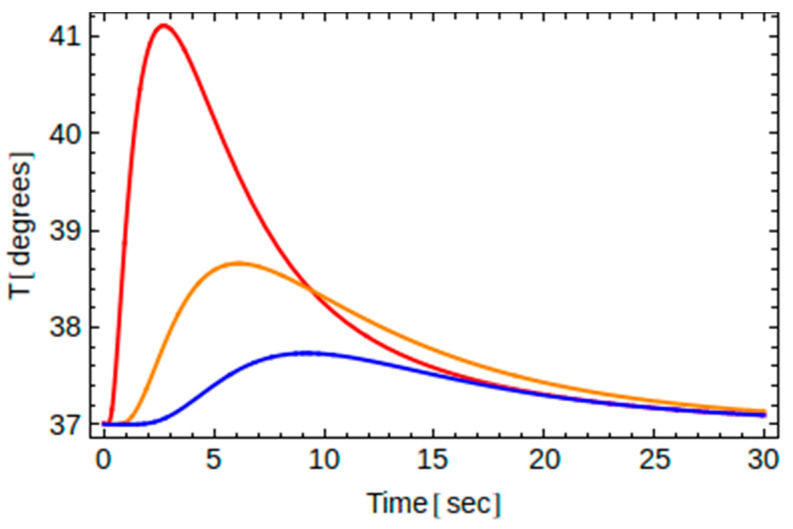
Temperature changes (in degrees °C) versus time (in sec) for implant A and for *t*_0_ = 2 s at three different locations, namely B1 (*y* = 3 *L*/4) in red, B2 (*y* = *L*/2) in orange, and B3 (*y* = *L*/4) in blue.

**Figure 5:**
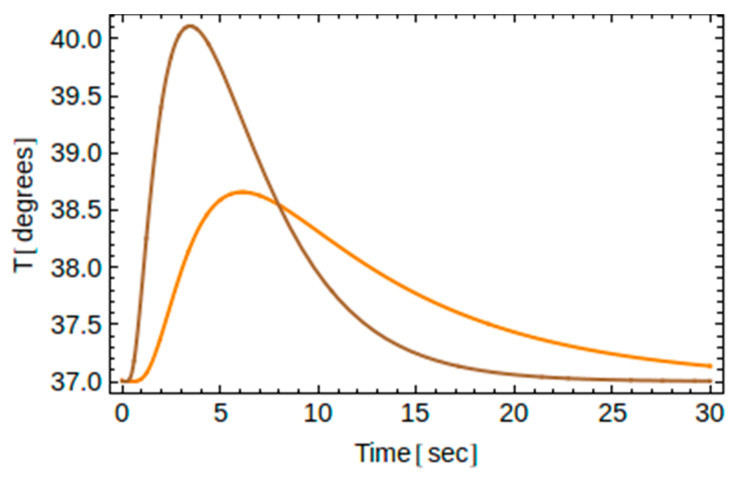
Temperature changes versus time at point B2 for implants A (orange) and B (brown) and for *t*_0_ = 2 s.

**Figure 6:**
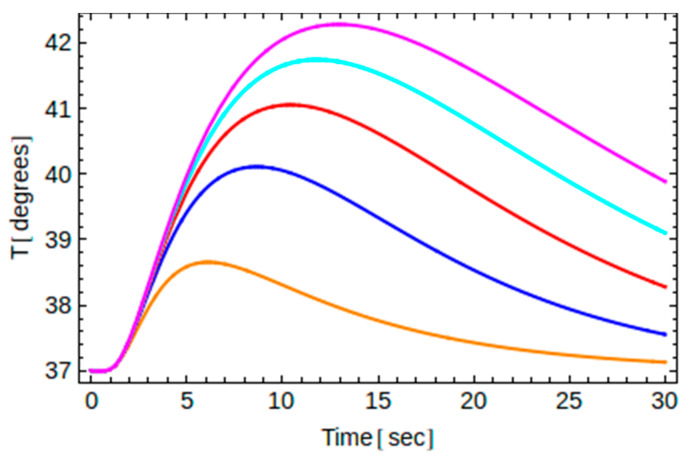
Temperature changes versus time at point B2 for implant A (=8.6 s) and for *t*_0_ = 2 s, 5 s, 8 s, 11 s, and 14 s from bottom to top.

**Figure 7:**
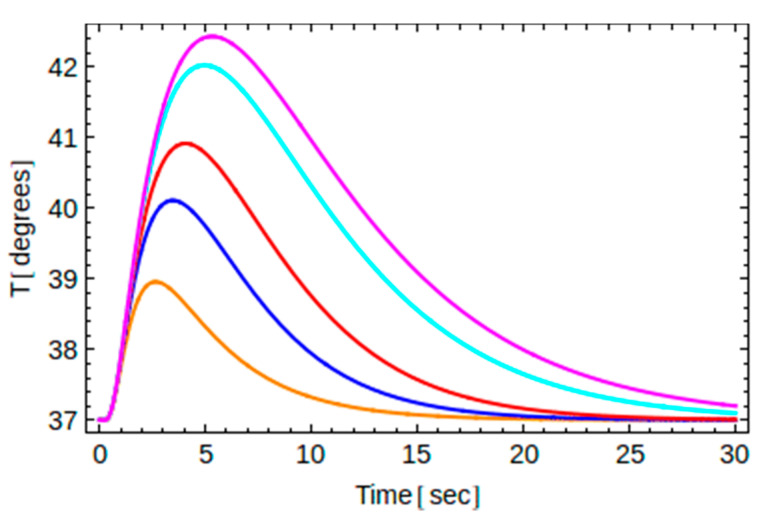
Temperature changes versus time at point B2 for implant B (=3.4 s) and for *t*_0_ = 1 s, 2 s, 3 s, 5 s, and 6 s from bottom to top.

The main features of the obtained analytic solution for the implants A and B as well as for several values of the exposure time are shown graphically in Figure 3, Figure 4, Figure 5, Figure 6 and Figure 7.

Recall that for a given thermal load (i.e., known *t*_0_) and for a given implant material (i.e., known thermal diffusivity *α*), the temperature depends on two independent variables, namely, one one hand, on the time *t*, and on the other hand, on the location point *y* along the axis of the implant. Therefore, one may plot *T* versus *t* for a certain point *y*, or plot *T* versus *y* at a given instant of time. This is shown in Figure 3 and Figure 4 below.

First, to see how the temperature varies along the implant at a given instant of time, in Figure 3, we show the temperature distribution from *y* = 0 up to *y* = *L* at four different instants of time, *t* = 9, 12, 15, and 18 s, for implant A and for *t*_0_ = 2 s. At every instant of time, the temperature at the end points remains the same due to the imposed boundary conditions, while at a certain location, i.e., fixed y point, the temperature decreases with time.

The impact on the temperature of the location point along the implant is shown in Figure 4, where we show that the temperature varies with time at points B1, B2, and B3 for implant A and *t*_0_ = 2 s. As we go deeper, the maximum temperature reached decreases and the time needed to reach it increases. The highest temperature is observed at point B1 due to its proximity to the thermal load.

To see the impact of the material chosen on temperature changes, in Figure 5, we show temperature changes for both implants A and B at point B2 for *t*_0_ = 2 s. The good conductor (type B in brown) reaches the highest temperature fast, while the moderate conductor (type A in orange) reaches a lower highest temperature later, due to the fact the heat is transferred slower in the case of implant A. Our results shown in Figure 3 and Figure 4 have been also observed in [25].

Finally, in Figure 6 and Figure 7, we show temperature changes at point B2 for five different exposure times *t*_0_ for implants A and B, respectively. In particular for implant A, with an intrinsic time of 8.6 s, we have considered *t*_0_ = 2, 5, 8, 11, and 14 s, from bottom to top, while for implant B, with an intrinsic time of 3.4 s, we have considered *t*_0_ = 1, 2, 3, 5, and 6 s, from bottom to top. For both implants, the curve in the middle corresponds to the case where the exposure time is very close to the intrinsic time of the implant. We see that the highest temperature observed increases with the exposure time both for implant A and implant B. Note that when the exposure time approaches the intrinsic time of the implant, the highest temperature reached is approximately 41 degrees, just below the critical temperature of 42 degrees, irrespectively of the material chosen.

A typical implant system is made of Titanium and metal alloys (fixture); hence, a continuous thermal conduction pathway (and heat reservoir) is created between the oral cavity and deeper parts of the jawbone. Herein, heat conduction is mainly mitigated by implant design and diameter (and time). The temperature at the abutment–implant interface is vital. The same is true for the type and amount of metal in the implant composition, whereby in situ heat transfer to supporting per-implant tissues can be significantly accelerated. Therefore, to reduce risks of injury or damage, the exposure time should be lower than the intrinsic time, or, if it is higher, it must be as close to the “proper” time as possible. Clinicians should minimize heat generation during implantology procedures and advise patients to restrain from or avoid hot beverages as much as possible until satisfactory clinical stability or even full osseointegration are evident, especially in cases of delayed implant loading/restoration.

Figure 2, Figure 3, Figure 4, Figure 5, Figure 6 and Figure 7 have been produced by employing a Wolfram (wolfram.com) Computing (accessed principally on Wednesday, 30 January 2019) Mathematica 12.30 file.

## 4. Conclusions

**Figure 8:**
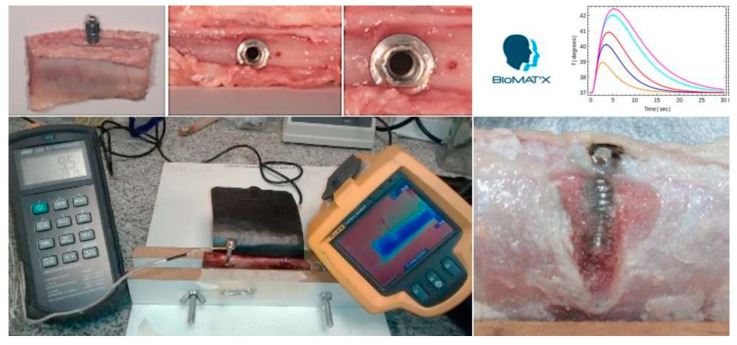
Experimental Set-up; developed in-House (at the BioMAT’X R&D&I Laboratory—Haidar Lab, CiiB, Faculties of Medicine and Dentistry, Universidad de los Andes, Santiago de Chile) as an ex-vivo heat distribution model employing human patient-grade titanium dental implants placed into porcine ribs (without coolant) and thermal changes monitored/recorded (quantified) using a CorDEX TP3R ToughPix DigiTherm Digital Thermal Camera (*an ongoing investigation*).

The cornerstone of successful dental implant therapy is osseointegration. Despite the fact that dental implants are a predictable (and preferred) treatment modality for the rehabilitation of partially and completely edentulous patients, high temperatures may cause irreversible damage to surrounding tissues and organs, with undesirable outcomes and sequels. In this work, to summarize, we have addressed the interesting problem of transient heat transfer and temperature changes in titanium dental implants upon hot liquid intake. To that end, we have solved the heat equation with appropriate initial and boundary condition assuming an exponential thermal load modeling the consumption of hot beverages. We have obtained an exact analytical solution, filling a gap in the literature, since, to the best of our knowledge, it was something that was missing. We have investigated what the impact of the material chosen, the location point along the implant, and the exposure time of the thermal load are on temperature changes. Furthermore, we have introduced in this work, for the first time, the intrinsic time that characterizes the geometry of the dental implant, and we have shown graphically how the interplay between this “intrinsic” time and the exposure time of the thermal load influences temperature changes. We conclude that the exact analytical solution obtained here, despite its simplicity, encapsulates all the physics, and it nicely reproduces the key features previously obtained in other numerical analyses. Thermal stress should not be ignored in evaluating the performance of metal dental implants [34,35,36]. This work can benefit the dental implant manufacturer, dental diagnostic (accurate computed tomography scanning) industry, clinical operator, and the patient, consequently, via considering or controlling intra-oral temperatures and minimizing or preventing peri-implant tissue(s) damage and the onset of osteotomy-related side effects; this would therefore complement the overall treatment plan for an enhanced dental implant stability, free of the undesirable interference (osseointegration at the local cellular level) at the bone–implant interface, whether for immediate or delayed loading strategies (in various bone-types/conditions) [34,35,36,37]. It is worth mentioning here to consider shorter dental implants (such as 4.0 × 4.0 mm) as an alternative to longer fixtures [38]. Perspective: Our continuing work seeks to validate our results in a laboratory-based ex vivo heat distribution model (in-House) employing osseointegrated human patient-grade Titanium dental implants that have been placed into porcine ribs (without coolant), followed by monitoring thermal changes (recorded and then plotted, quantifiably, under various conditions) using a CorDEX TP3R ToughPix DigiTherm Digital Thermal Camera, as is shown in our experimental set-up in Figure 8, a currently-ongoing investigation at our BioMAT’X Lab.

## Data Availability

The data presented in this study are available in Figure 2, Figure 3, Figure 4, Figure 5, Figure 6 and Figure 7 and Appendix A  and Appendix B. Raw data presented in this study are also available on request from the corresponding author. The data are not publicly available due to ongoing experimental work as in Figure 8, which are planned to be published in a sequel article, upon commencing the patenting process(es).

## Appendix A

The heat equation can be derived using energy conservation, Fourier’s law for the heat flux, *q* = −*k* Δ*T*, with k being the thermal conductivity of the material, and the expression for heat, Q = m *c T*, where *c* is the specific heat of the material. In the one-dimensional case, applying energy conservation in an infinitesimal volume A Δ*x* with mass *ρ* A Δ*x*, where ρ is the mass density, we can see that during an infinitesimal time interval Δ*t*, the heat inside the volume changes according to Fourier’s law:

(A1) $$c\rho \frac{\Delta T}{\Delta t}=k\frac{\frac{\partial T}{\partial x}\left(x+\Delta x\right)-\frac{\partial T}{\partial x}\left(x\right)}{\Delta x}$$

and taking the limit Δx→0 we finally obtain the heat equation

(A2) $$\frac{\partial T}{\partial t}=\alpha \frac{\partial^{2}T}{\partial x^{2}}$$

where the thermal diffusivity is given by *α* = *k*/(*c ρ*). For a given material, one can find in tables the values of *k*, *c*, and *ρ*, and then using the previous simple formula, one can compute *α* for that material. Typically, in the International System of units (S.I.), the mass density is of the order of 1000 (kg $\begin{array}{c} m^{-3} \end{array}$), the thermal conductivity of the order (1–10) J per (m s °C), and the specific heat of the order of 1000 J per (kg °C) (see e.g., Table 1 of [22,23]). Therefore, the thermal diffusivity turns out to be ($10^{-6}$–$10^{-5}$) ($\begin{array}{c} m^{2} \end{array}$/s). For example, for titanium, *α* = 9.0 × $10^{-6}$($\begin{array}{c} m^{2} \end{array}$/s) [39], while for ceramic, *α* = 6.2 × $10^{-7}$($\begin{array}{c} m^{2} \end{array}$/s) [40].

## Appendix B

We briefly summarize how to solve an ordinary, inhomogeneous first order linear differential equation. First, we find the solution of the corresponding homogeneous equation without the source term, and then, we find a particular solution that satisfies the non-homogeneous equation.

Thus, if the equation has the form

(A3) ż+az=Γexp(−λt)

with the initial condition *z*(0) = *z*0, then the solution of the corresponding homogeneous equation is given by *zo*(*t*) = *C exp*(−*at*), where *C* is an arbitrary constant. To obtain the partial solution, we assume that it is of the same form as the source term, namely *zp*(*t*) = Δ *exp*(−*λ t*), and by incorporating it into the equation, we find that Δ = Γ/(*α* − *λ*). Taking the initial condition into account, the final expression for the solution is given by:

(A4) $$z\left(t\right)=\left(z_{0}+\frac{\Gamma }{\lambda -a}\right)exp\left(-at\right)+\frac{\Gamma }{a-\lambda }exp\left(-\lambda t\right)$$

To obtain the above solution, it is assumed that the parameters *a* and *λ* are different. However, in the special case in which they coincide, the partial solution of the differential equation

(A5) ż+az=Γexp(−at)

is *zp*(*t*) = Γ *t exp*(−*a t*), and thus the full solution is given by:

(A6) $$z\left(t\right)=\left(z_{0}+\Gamma t\right)exp\left(-at\right)$$
